# Sulfated modification and anti-tumor activity of laminarin

**DOI:** 10.3892/etm.2013.1277

**Published:** 2013-08-29

**Authors:** CHEN-FENG JI, YU-BIN JI, DE-YOU MENG

**Affiliations:** 1 Engineering Research Center of Natural Anticancer Drugs, Ministry of Education, Harbin University of Commerce, Harbin, Heilongjiang 150076, P.R. China; 2 Center of Research on Life Science and Environmental Science, Harbin University of Commerce, Harbin, Heilongjiang 150076, P.R. China

**Keywords:** laminarin, sulfated, modification, antitumor activity

## Abstract

The aim of this study was to investigate the sulfated modification of laminarin and the changes in structure and antitumor activity. The chlorosulfonic acid-pyridine method was applied for sulfated modification. The molecular weights of laminarin and laminarin sulfate (LAMS) were measured by high-performance liquid chromatography (HPLC), and IR and NMR spectra were also recorded. The surface conformations of laminarin and LAMS were observed with a scanning electron microscope. The antitumor activities of the two polysaccharides were also evaluated using an MTT assay. LAMS with a sulfate content of 45.92% and a molecular weight of 16,000 was synthesized. The IR spectra of laminarin and LAMS showed the characteristic absorption peaks of a polysaccharide, and LAMS also had the characteristic absorption peaks of sulfate moieties. The NMR spectra showed that laminarin and LAMS had β-(1→3) glycosidic bonds forming the main chain, and sulfate substitution was at the hydroxyl groups of C_2_ and C_6_. Under the scanning electron microscope, there were clear differences in surface conformation between laminarin and LAMS; laminarin was cloud-like and spongy, while LAMS was block-like and flaky. The MTT results showed that laminarin and LAMS had inhibitory effects on LoVo cell growth, and the antitumor activity of LAMS was higher than that of laminarin at the same concentration. This suggests that sulfated modification was able to change the laminarin structure and markedly enhance the antitumor activity.

## Introduction

Polysaccharides are macromolecules that are able to transfer large amounts of biological information between cells (1). The biological activities of polysaccharides may be developed and enhanced by means of chemical modifications, which broaden their application and clinical use. Sulfated modification may produce high activity, good functional polysaccharides and polysaccharide derivatives, and the introduction of sulfate groups may change the physicochemical properties, three-dimensional conformation and activities of the polysaccharides (2,3). Previous studies revealed that certain polysaccharides without antitumor activity showed antitumor activity following sulfated modification, while certain sulfated polysaccharides with antitumor activity demonstrated a reduction or loss of antitumor activity following the removal of sulfate groups (4,5). The main methods used for the sulfated modification of polysaccharides include the sulfuric acid method, chlorosulfonic acid-pyridine method, chlorosulfonic acid-carboxamide method, sulfur trioxide-pyridine method and sulfur trioxide-dimethylformamide method. The chlorosulfonic acid-pyridine method is widely used due to the high yields and degree of substitution of the products (6,7).

Laminarin, also known as brown algae starch, is an active component in kelp that shows numerous activities, including antitumor, immunomodulatory, antibacterial, antiviral, blood lipid regulating, anti-oxidation, anticlotting, antiradiation and hypoglycemic activities (8,9). Ji *et al* (10) reported that laminarin was able to inhibit LoVo human colon cancer cell proliferation and induce LoVo cell apoptosis through a mitochondrial pathway. In the present study, we selected to modify laminarin by the chlorosulfonic acid-pyridine method, and then investigated the differences in the structures and antitumor activities of laminarin and laminarin sulfate (LAMS). This may provide a scientific basis for further studies concerning polysaccharide modifications and the comprehensive developments and utilizations of polysaccharides.

## Materials and methods

### Main reagents

Laminarin, dextrans, MTT and DMSO were purchased from Sigma-Aldrich (St. Louis, MO, USA). The chlorosulfonic acid, pyridine, trichloroacetic acid (TCA), potassium sulfate (K_2_SO_4_), gelatin, barium chloride and dimethylformamide (DMF) were purchased from Tianjin Kemiou Chemical Reagent Co., Ltd. (Tianjin, China). DMEM/F12 culture medium was purchased from Thermo Scientific (Waltham, MA, USA). Fetal bovine serum was purchased from Hangzhou Sijiqing Biological Engineering Materials Co., Ltd. (Hangzhou, China). Trypsin was purchased from Invitrogen (Carlsbad, CA, USA).

### Main apparatus

The UV1000 UV-VIS spectrophotometer was purchased from Techcomp Ltd. (Shanghai, China), The 2695 HPLC system was purchased from Waters (Milford, MA, USA), the FTS-3100 FT-IR spectrometer was purchased from Varian Medical Systems, Inc. (Salt Lake City, UT, USA), the AVANCE III 400 MHz spectrometer was purchased from Bruker (Karlsruhe, Germany), the QUANTA 250 FEG scanning electron microscope was purchased from FEI (Hillsboro, OR, USA), the Model 680 microplate reader was purchased from Bio-Rad (Hercules, CA, USA) and the CB-150 CO_2_ incubator was purchased from New Brunswick Scientific (Edison, NJ, USA).

### Sulfated modification of laminarin

Pyridine (3 ml) was added to a bottle and cooled in an ice-bath. Chlorosulfonic acid (1 ml) was slowly dropped into the pyridine solution. After 1 h, a primrose-yellow esterification product appeared and the bottle was removed from the ice-bath. Laminarin (100 mg) was dissolved in 10 ml dimethylformamide (DMF) and agitated with a magnetic stirrer for 20 min. The mixture was added to the esterification product bottle. The bottle was placed into a hot water bath (75°C) for 1.5 h. Then, the bottle was cooled and 25 ml ice-water was added. The solution was adjusted to pH 7.0 with 2.5 mol/l NaOH and 75 ml ethanol was added. The solution was centrifuged, and the sediment was dissolved in water and dialyzed with distilled water for 3 days. The solution was concentrated and freeze-dried to provide LAMS.

### Preparation of sulfate standard curve

K_2_SO_4_ (108.75 mg) was weighed and dissolved in 1 mol/l hydrochloric acid to a volume of 100 ml. K_2_SO_4_ solution (0.0, 0.08, 0.16, 0.24, 0.32 and 0.40 ml) was extracted with a pipette and made up to a final volume of 0.40 ml with 1 mol/l hydrochloric acid in a test tube. TCA (7.6 ml, 3% w/v) and 2.0 ml barium chloride (1% w/v, 10 g/l) and gelatin (0.5% w/v, 5 g/l) solution were added to the test tubes separately. After 15 min the absorbance of each solution was determined at λ=360 nm with a UV1000 UV-VIS spectrophotometer and the absorbance value A1 was obtained. Gelatin solution (2.0 ml; 0.5% w/v, 5 g/l), instead of barium chloride-gelatin solution, was added to the test tubes and the absorbance value A2 was determined at λ=360 nm. A standard curve was drawn with the sulfate concentration (*μ*g/ml) and A1-A2 as horizontal and vertical coordinates, respectively.

### Determination of sulfate content in LAMS

LAMS (5 mg) was accurately weighed and dissolved in 5 ml 1 mol/l hydrochloric acid. The resulting solution was incubated in a water bath at 100°C for 6 h. The cooled solution was put into a 5-ml volumetric flask and diluted with 1 mol/l hydrochloric acid to the 5-ml mark. A 0.4-ml sample of the solution was removed and placed into a test tube. TCA (7.6 ml, 3% w/v) and 2.0 ml barium chloride (1% w/v, 10 g/l) and gelatin (0.5% w/v, 5 g/l) solution were added to the test tubes separately. After 15 min the absorbance of each solution was determined at λ=360 nm with a UV1000 UV-VIS spectrophotometer and the absorbance value A1 was obtained. Gelatin solution (2.0 ml; 0.5% w/v, 5 g/l), instead of barium chloride-gelatin solution, was added to the test tubes and the absorbance was measured at λ=360 nm to determine A2. The concentration of sulfate in the samples was calculated according to a linear regression equation.

### Measurement of molecular weight

The molecular weight was measured by high high-performance liquid chromatography using the 2695 system with a refractive index detector. The column and the detector were maintained at 40°C. The mobile phase was 0.2 mol/l ammonium acetate buffering at a flow rate of 0.8 ml/min. Standard dextrans (2 mg) were dissolved in 1 ml 0.2 mol/l ammonium acetate. The dextrans were measured by HPLC and their retention time was recorded. A standard curve was drawn with the logarithm of standard dextrans molecular weight (lgMW) and retention time (tR) as horizontal and vertical coordinates, respectively. Laminarin and LAMS (2 mg) were dissolved in 1 ml 0.2 mol/l ammonium acetate, measured by HPLC and their retention time was recorded. The molecular weights of laminarin and LAMS were calculated according to a linear regression equation.

### IR spectra

The sample (5 mg) and KBr (400 mg) were homogenized for 5–10 min, pressed into a tablet and then scanned at wavelengths of 4,000-400 cm^−1^ with a FTS-3100 FT-IR spectrometer.

### NMR spectra

The sample (10 mg) was dissolved in 1.0 ml D_2_O, followed by centrifugation and lyophilization. The process was repeated twice and the final sample was dissolved in 0.5 ml D_2_O. The ^1^H and ^13^C NMR spectra were recorded on an AVANCE III 400 spectrometer at 25°C.

### Observation under scanning electron microscope

The sample powder was fixed on a conductive adhesive. The three-dimensional images were produced by a QUANTA 250 FEG-scanning electron microscope at 20 kV.

### LoVo cell culture

The LoVo human colon cancer cell line was provided by The Center of Research and Development on Life Sciences and Environmental Sciences of Harbin University of Commerce (Harbin, China). The LoVo cells were cultured in DMEM/F12 medium containing 10% fetal bovine serum at 37°C in a 5% CO_2_ humidified incubator. Research carried out on human cells followed international and national regulations. The Biomedical Research Ethics Committee in the Engineering Research Center of Natural Anticancer Drugs, Ministry of Education of China (Harbin, China) had approved the experiments undertaken.

### Antitumor activity

The exponentially growing cells were washed, digested with trypsin and resuspended in DMEM/F12 medium to a concentration of 1×10^5^ cells per ml. In a 96-well plate, 100 *μ*l cell suspension per well was cultured for 24 h. The cells were then incubated with varying concentrations of laminarin or LAMS (400, 800 or 1,600 mg/l) for a further 72 h with 12 wells per concentration. The cells were incubated with 100 *μ*l DMEM/F12 culture medium only as a control group. MTT was dissolved in PBS to provide a solution with a final concentration of 0.5 mg/ml. After 72 h, the cell suspension was discarded, then 200 *μ*l of the 0.5 mg/ml MTT solution was added into each well and incubated at 37°C for 4 h. All media were then removed and 150 *μ*l DMSO was added to each well to dissolve the purple formazan crystals. The plate was agitated for 3 min and the spectrophotometric absorbance at 570 nm was read using a Model 680 microplate reader. The inhibition rate was used to evaluate the cytotoxicity.

### Statistical analysis

Statistical comparisons within groups were carried out by one way ANOVA. P<0.05 was considered to indicate a statistically significant result.

## Results

### Sulfate content of LAMS

The linear regression equation of the sulfate standard curve was A=2.2856C-0.0205 (R^2^=0.999) and the content of SO_4_^2−^ showed a positive linear correlation in the range of 12–120 mg. According to the formula: f=W/C, the conversion factor f was 0.969. The content of SO_4_^2−^ in LAMS was 45.92%, and according to the formula: DS=(1.62×S%)/(32–1.02×S%), where W = weighing quality of sulfate, C = calculated quality of sulfate, S% = the content of sulfur (15.3% in LAMS). The substitution degree (DS) was 1.51.

### Molecular weight

The linear regression equation of the molecular weight standard curve was calculated as: lgM_W_=−0.787tR+12.542 (R^2^= 0.993), and the molecular weights of laminarin and LAMS were calculated to be 5,000 and 16,000, respectively.

### IR analysis

**Figure 1.**
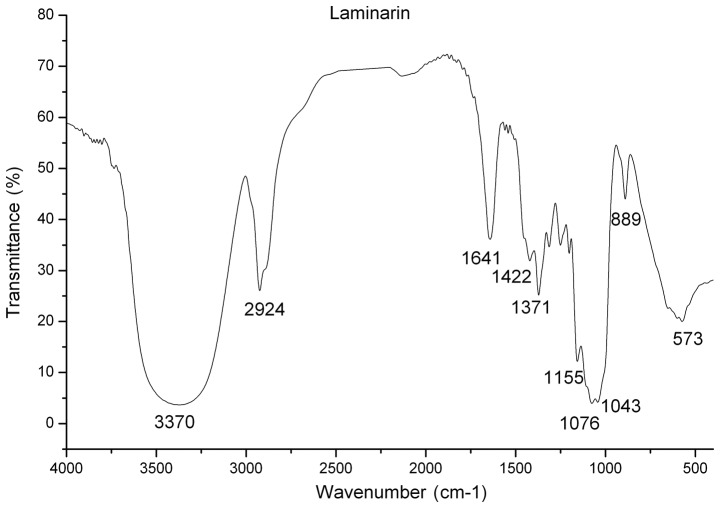
IR spectrum of laminarin.

**Figure 2.**
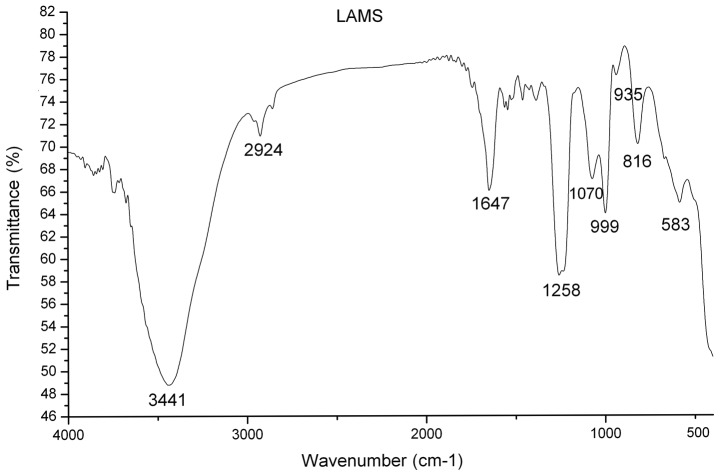
IR spectrum of LAMS. LAMS, laminarin sulfate.

The IR spectrum of laminarin showed the characteristic absorption peak of -OH stretching vibration at 3,370 cm^−1^ and the peak of C-H stretching vibration in -CH_3_ or -CH_2_ groups appeared at 2,924 cm^−1^, which are the characteristic absorption peaks of sugar. The characteristic pyranose absorption peaks were also visible. The characteristic absorption peak of C-H scissor vibration appeared at 889 cm^−1^, which indicated that the glycosidic bond in laminarin was β type. Therefore, the laminarin had a pyranose skeleton with a β-glucosidic bond. These results are shown in Fig. 1 and Table I. By comparison, the IR spectrum of LAMS showed the characteristic absorption peaks of S=O and C-O-S stretching vibrations at 1,258 cm^−1^ and 816 cm^−1^, respectively, which indicated that the sulfates were introduced into the sugar molecules. The IR spectrum also showed that LAMS has a pyranose skeleton with a β-glucosidic bond. The IR data for LAMS are shown in Fig. 2 and Table I.

### NMR analysis

The ^1^H-NMR spectrum of laminarin showed two absorption peaks in the range of δ4.5–5.5 ppm; one was from D_2_O and the other was from the anomeric hydrogen of glucose. Since the chemical shift of the anomeric hydrogen was δ4.758 ppm, which was <5.0 ppm, this indicated the glycosidic bond in laminarin was β type. The ^1^H-NMR spectrum of LAMS showed that the chemical shifts of hydrogen generally moved downfield, which indicated that some of the hydroxyl groups in laminarin had been sulfated. The spectrum also showed that LAMS had β-glycosidic bonds. The ^13^C-NMR spectrum of laminarin showed that the chemical shifts of C1 and C3 had moved downfield compared with those of glucose, indicating that the glucosidic bond in laminarin was β-(1→3) type. The ^13^C-NMR spectrum of LAMS showed that the chemical shifts of C2 and C6 had moved downfield and the peak intensity had weakened, whereas the chemical shifts of C1 and C3 had moved upfield, and the chemical shifts of C4 and C5 showed no change, thus indicating that the substitution positions of the sulfate groups were the hydroxyl groups of C2 and C6. Laminarin and LAMS have a pyranose skeleton with β-(1→3) glucosidic bonds, as shown in Table II.

### Conformation observation

**Figure 3.**
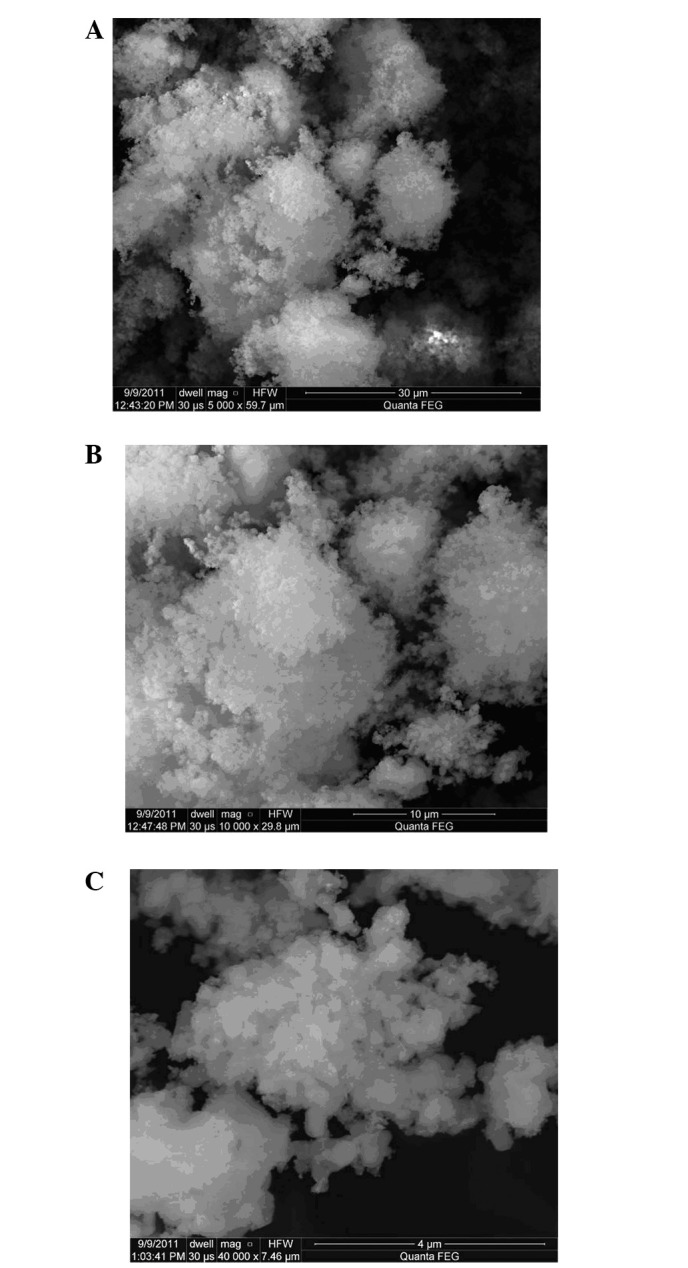
SEM images of laminarin. (A) Magnification, ×5,000; (B) magnification, ×10,000; (C) magnification ×40,000. SEM, scanning electron microscopy.

**Figure 4.**
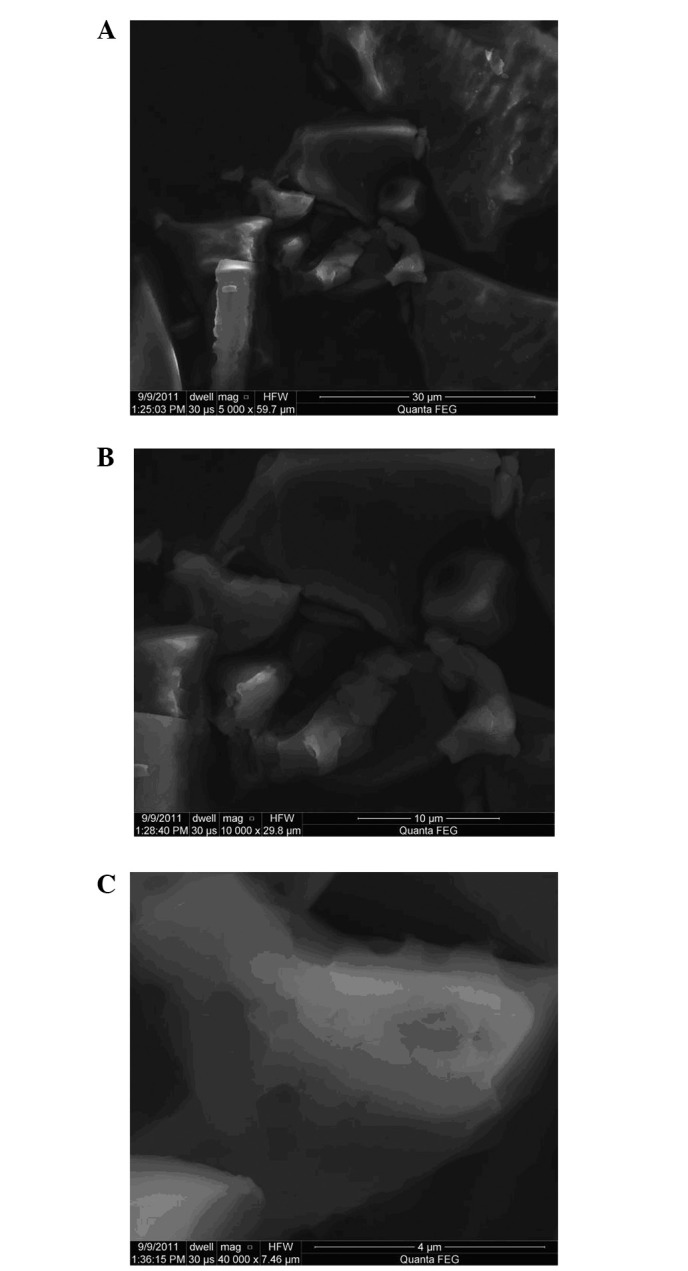
SEM images of LAMS. (A) Magnification, ×5,000; (B) magnification, ×10,000; (C) magnification ×40,000. SEM, scanning electron microscopy; LAMS, laminarin sulfate.

Under a scanning electron microscope, marked differences were observed in surface structure between laminarin and LAMS. Laminarin was spongy and cloud-like, and contained numerous dispersed sugar particles. LAMS was flakey and block-like, and contained few dispersed sugar particles, as shown in Figs. 3 and 4, respectively.

### Antitumor activity

After treatment with various concentrations of laminarin and LAMS for 72 h, MTT assay results showed that the two compounds inhibited LoVo proliferation. The inhibitory effects were significantly different from that in the control group (P<0.05) and concentration-dependent. After sulfated modification, the antitumor activity of laminarin was enhanced, and the inhibition rate of LAMS was significantly greater than that of the same concentration of laminarin (P<0.01; Table III).

## Discussion

Numerous pharmacological experiments have shown that certain polysaccharides have direct cytotoxic activity against tumor cells and directly kill cancer cells *in vitro*; the majority of polysaccharides have an antitumor effect *in vivo*, which enhances the activity of T and B lymphocytes, macrophages, natural killer (NK) cells and other immune cells, activates the complement system and promotes the production of cytokines, which then regulates the immune system (10,11).

Sulfated polysaccharides are polysaccharide derivatives in which the hydroxyl groups in the monosaccharide moieties of the polysaccharide chains are substituted by sulfate groups. These polysaccharides have complex chemical structures and biological activities. Through the induction of apoptosis, inhibition of cell proliferation, inhibition of tumor angiogenesis, regulation of immune function and improvement of the effects of chemotherapy drugs on tumor cells, the sulfated polysaccharides may be effective in cancer therapy. The biological activities of sulfated polysaccharides are closely associated with their structures and physicochemical properties. The steric and electrostatic repulsion effects of sulfate groups may change the spatial structure of the polysaccharide and the flexion of the sugar chain, thus increasing water-solubility and leading to changes in biological activity (12–14).

In the current study, LAMS with a substitution degree of 1.5 was synthesized by the chlorosulfonic acid-pyridine method. The IR spectrum of LAMS showed that the characteristic absorption peak of -OH weakened (3,441 cm^−1^), and the characteristic symmetric stretching vibration peak of C-O-S (816 cm^−1^) and the characteristic asymmetric stretching vibration peak of S=O (1,258 cm^−1^) appeared. The ^1^H-NMR spectrum of LAMS showed that the chemical shift of the hydrogen generally moved downfield, which indicated that some of the hydroxyl moieties in laminarin were sulfated. The ^13^C-NMR spectrum of LAMS showed that the chemical shifts of C2 and C6 moved downfield (by 3 and 6.5 ppm, respectively) and the peak intensity weakened, while the chemical shifts of C1 and C3 moved upfield, and the chemical shifts of C4 and C5 showed no change compared with those of laminarin, thus indicating that the substitution positions of the sulfate group were the hydroxyl groups of C2 and C6. Laminarin and LAMS both have a pyranose skeleton with a β-(1→3) glucosidic bond. Under a scanning electron microscope, there were clear differences in surface structure between laminarin and LAMS. Laminarin was spongy and cloud-like, whereas LAMS was block-like and flaky. Changes of shape indicate a chemical change in the majority of cases. In LAMS, the substitution of the hydroxyl groups of the sugar units by sulfate groups may lead to a change in the conformation of the sugar chain, and repulsion between sulfuric acid groups may result in a conformation showing extended or rigid structure.

Colon cancer is a common malignant tumor in the digestive tract and one of the four most common malignant tumors throughout the world. In a previous study (10), laminarin was shown to be a potent agent for cancer prevention and inhibited LoVo human colon cancer cell proliferation. The antitumor experiment showed that after sulfated modification, the anti-tumor activity of laminarin was significantly enhanced, and the inhibition rate of LAMS was significantly greater than that of laminarin at the same concentration. This may be due to the sulfated modification changing the molecular structure and spatial conformation of the polysaccharide, leading to changes in biological activity. When the hydroxyl group of a laminarin sugar unit is substituted by a sulfate group, the conformation of the sugar chains distorts or changes, and the formation of a non-covalent bond becomes easier. In addition, repulsions between the anionic groups elongate the sugar chain, and some of the sulfuric acid and hydroxyl groups on the sugar chain may form hydrogen bonds. This may result in the chain forming a helical structure and adopting an active conformation, thus causing the increase in its activity.

Sulfated modifications may help to produce high activity, good functional polysaccharides and polysaccharide derivatives. How the introduction of sulfate moieties affects the properties and conformation of the polysaccharide, and thus affects the biological activity, has yet to be investigated. Thus, it is necessary to carry out in-depth studies in order to investigate polysaccharide structure-activity relationships, which may provide a theoretical basis for polysaccharide research and development.

## Figures and Tables

**Table I.** IR analysis of laminarin and LAMS.

Group	Vibration mode	Peak (cm^−1^)	
		Laminarin	LAMS
O-H	O-H stretching vibration	3370	3441
-CH_2_-	C-H stretching vibration	2924	2978
C=O	Symmetric and asymmetric stretching vibration	1641	1649
C-O	C-O stretching vibration	1043, 1076	1070
-O-SO_3_	S=O stretching vibration	-	1258
-O-SO_3_	C-O-S stretching vibration	-	816

LAMS, laminarin sulfate.

**Table II.** Change of chemical shifts of laminarin after sulfation.

Sugar carbon	Chemical shift (ppm)	
	Laminarin	LAMS
C1	102.5	101.2
C2	75.6	77.9–78.7
C3	84.2	83.0
C4	68.1	67.7
C5	73.3	72.7–73.4
C6	60.7	67.0

LAMS, laminarin sulfate.

**Table III.** Inhibitory effects of laminarin and LAMS on LoVo cells by MTT assay.

Samples	Concentration (*μ*g/ml)	OD (mean ± SD)	IR (%)
Control	0	0.923±0.065	-
Laminarin	400	0.727±0.053^a^	21.24
	800	0.674±0.061^b^	26.98
	1600	0.564±0.072^b^	38.89
LAMS	400	0.247±0.055^bc^	73.24
	800	0.184±0.028^bc^	80.06
	1600	0.124±0.042^bc^	86.57

a P<0.05 or

b P<0.01 compared with the control;

c P<0.01 compared with laminarin. LAMS, laminarin sulfate; OD, optical density.
